# Supplementary material for: EnzML: multi-label prediction of enzyme classes using InterPro signatures
Source: BMC Bioinformatics. 2012 Apr 25;13:61. doi: 10.1186/1471-2105-13-61 (PMC3483700; doi:10.1186/1471-2105-13-61)
Supplement: Addtional file 5 — The Java code to format the data files, evaluate and predict. The file enzml_java_code.tar.gz contains the Java code used to format database data to ARFF and XML formats, to execute cross and train-test (jackknife) evaluations and to record evaluation results to database. More information is included in the readme.txt file and the Javadoc files. The code can be used with a MySQL database. To use a different database software, other JDBC drivers might be required. [file 1471-2105-13-61-S5.gz › java_code/utils/doc/uk/ac/ed/inf/utils/database/package-summary.html]

uk.ac.ed.inf.utils.database


---


|  |  |  |
| --- | --- | --- |
| **Overview** \| **Package** \| Class \| **Use** \| **Tree** \| **Deprecated** \| **Index** \| **Help** | |  |
| **PREV PACKAGE**   **NEXT PACKAGE** | **FRAMES**    **NO FRAMES**     **All Classes** |  |


---

## Package uk.ac.ed.inf.utils.database

| **Class Summary** | |
| --- | --- |
| **DbConn** | A DbConnectionProperties object contains the properties needed to connect to a database through JDBC. |
| **DbCreator** | Class |
| **DbManaged** | Managed is a class which has a `DbManager` (an object that coordinates its database activities) |
| **DbManager** | A database manager provides object to create a database, write and read from it. |
| **DbReader<T,U>** | Class to read from a database. |
| **DbUtils** | Database utilities (to create sql code etc.) |
| **DbWriter** | To write to a database table |
| **Managed** | A class with a manager object |
| **Manager** | A manager manages: for example, for a database object, the `DbManager` manages a `DbCreator` to create the tables, `DbReader` to read data from and `DbWriter` to write data to the database. |
| **ResultSetUtils** | Class |
| **SqlUtils** | Class |
| **Table** | An SQL database table definition (table name + columns) |
| **TableColumn** | A column object contains all the information to create, fill and delete a database table column |
| **TableCreator** | Class to create a table object in the database. |
| **TableManaged** | TableManaged is a class which has a `TableManager` (an object that coordinates its table activities) |
| **TableManager** | Class to create/read/write to a database table. |
| **TableReader** | Class |
| **TableRow** | Contains the data for a generic row (a list of [table column, value] entries) to be written to a database table. |
| **TableWriter** | A TableWriter object contains all the information to create, fill and delete a database table. |

---


|  |  |  |
| --- | --- | --- |
| **Overview** \| **Package** \| Class \| **Use** \| **Tree** \| **Deprecated** \| **Index** \| **Help** | |  |
| **PREV PACKAGE**   **NEXT PACKAGE** | **FRAMES**    **NO FRAMES**     **All Classes** |  |


---
